# The Influence of Freeze–Thaw Cycles and Corrosion on Reinforced Concrete and the Relationship between the Evolutions of the Microstructure and Mechanical Properties

**DOI:** 10.3390/ma15186215

**Published:** 2022-09-07

**Authors:** Shuhua Zhang, Bin Tian, Bofu Chen, Xiaochun Lu, Bobo Xiong, Ning Shuang

**Affiliations:** College of Hydraulic and Environmental Engineering, Three Gorges University, Yichang 443002, China

**Keywords:** concrete, freeze–thaw, steel bar corrosion, microstructure, bond strength

## Abstract

Freeze–thaw cycles (FTCs) and steel bar corrosion (SBC) are the most common service conditions of hydraulic concrete and have significant impacts on its durability. Using pullout and microscopic tests of different FTC and SBC rates, we selected the mass loss rate, ultrasonic velocity, bond strength and bond slip in order to describe the changes in the macro-properties, and also selected the porosity and pore size distribution as micro-parameters in order to explore the influence of FTCs and SBC on the mechanical properties of hydraulic concrete. The results showed that the bond strength decreased as the FTCs increased due to the microstructure damage caused by FTC and SBC, which affects the mechanical properties. A corrosion rate of ≤3% offset the damage caused by 50 FTCs. FTCs and SBC resulted in superimposed damage effects on the concrete. In addition, we established a bond strength damage model based on the joint FTCs and SBC and quantitatively described the degradation law of the macro-mechanical properties. The analysis shows that the influence of FTCs on the bond strength was greater than that of the SBC. These research results can provide a reference and experimental support for the frost-resistant design and durability prediction of hydraulic concrete structures in cold environments.

## 1. Introduction

Concrete structures in northern China encounter complex service environments during their service life [1]. The factors affecting the durability of concrete structures usually include freeze–thaw cycles (FTCs), steel bar corrosion (SBC) and external loads, which alone or alternately lead to the deterioration of the mechanical properties of concrete structures [2]. FTC damage is one of the main factors leading to the deterioration of existing reinforced concrete structures in cold areas and seriously affects the service life of concrete buildings [3,4,5,6]. Another major factor is SBC, which has attracted great attention in civil engineering [7]. Because of seasonal temperature changes, nearly 100% of reinforced concrete structures in Northeast China are affected by freeze-thaw damage [8]. Compared with other reinforced concrete structures, hydraulic-reinforced concrete is more prone to freeze-thaw and SBC in areas where there are changes in water levels due to long-term contact with reservoirs [9], particularly in the case of concrete face slabs in rockfill dams [10]. FTCs lead to microscopic changes in the pore structure and ion transport properties of the concrete, producing microcracks that may accelerate the ion transport and the corrosion rate of the steel reinforcement. The volume of corrosion caused by SBC in reinforced concrete is about two to six times that of the original steel bar, which exerts great pressure on the surrounding concrete. It eventually causes the concrete to crack, and the crack will gradually expand to the concrete surface [11]. FTC damage and SBC in hydraulic-reinforced concrete are common phenomena in cold areas [12,13]. The combined actions of the freeze–thaw cycle and reinforcement corrosion may exacerbate the degradation of the mechanical properties of the concrete, thus increasing the risks regarding the safety of the structure.

Sufficient bonding force is the basis for the capacity of the two materials, steel bar and concrete, to work together and coordinate deformation [14]. In recent years, numerous studies have been conducted on the bond degradation of concrete structures under FTC action [15]. A study by Tu et al. [16] examined the effects of FTCs on the bond performance of thermal insulation concrete, and CT scanning technology was used to study the evolution of the internal structure of the thermal insulation concrete subjected to FTCs. Shang et al. [17] studied the bond properties (failure mode, bond strength and bond stress–slip curve) between steel bars and recycled aggregate concrete after different FTCs. The tests investigated and analyzed the effects of the FTC time, steel bar type and deformed steel bar diameter on the bond performance between steel bars and recycled aggregate concrete. Ma et al. [18] found that FTCs damage concrete and accelerate the corrosion of steel bars. Wang et al. [19] studied the effects of FTCs and chloride ion erosion on steel bar corrosion in concrete from a macro point of view and considered that, although FTCs caused serious spalling in all specimens, the risk of steel bar corrosion was still in the “passive region.” Liu et al. [20] investigated the bond behaviors between deformed steel bars and recycled concrete subjected to FTCs. It was found that the bond strength decreased as the FTCs increased, and the effect of the freeze-thaw damage on the bond strength depended on the evolution of the microscopic properties of the reinforced concrete. Via loading, FTC and bond-slip tests, Ma et al. [21] studied the effects of freeze-thaw damage and continuous load coupling on the bond properties of various kinds of concrete. The results showed that freeze-thaw damage reduces the bond performance of concrete. In a study of the effects of corrosion on concrete durability, Zhang [22] studied the effects of the combined actions of FTC and chloride on steel bar corrosion. The results showed that freeze-thaw damage accelerated the penetration of chloride ions into the concrete and accelerated the corrosion of the steel bars. Wang [23] studied the influences of FTC and SBC on the flexural capacity of reinforced concrete beams by accelerated corrosion tests and rapid freeze–thaw cycle tests, with the yield to strength ratio as the test index. The finite element analysis showed that the corrosion level of the reinforcement had a great influence on the flexural performance of the reinforced concrete beams, and the reduction percentage of the flexural bearing capacity of the beams was directly related to the corrosion level of the reinforcement [24].

The abovementioned studies mainly focused on how the FTC influences the bond performance between the reinforcement and concrete (bond strength and bond slip) from a macro perspective, with less attention given to the influence of the joint actions of FTCs and SBC on the bond performance. A lower FTC, in the current research, may not be sufficient to explain the effects of greater TFC damage on the mechanical properties of reinforced concrete. In addition, because of the limited sample points selected for the microscopic experiments, many studies do not clearly describe the relationship between the macro-properties and microstructure, and there are few studies presenting bond strength damage models based on the number of FTCs and rates of SBC. In conclusion, further study of the influences of FTC and SBC on the macro-performance, on the microlevel, is necessary. An understanding of the damage mechanism of reinforced concrete structures under the combined actions of FTCs and SBC is also of great significance for the prediction of the durability of concrete and alterations to the corrosion resistance of reinforced concrete in cold environments. In this paper, the effects of FTCs and SBC on reinforced concrete and the relationship between the evolutions of the microstructure and mechanical properties are studied using the central pullout test, combined with the evolution of damage indices (e.g., mass loss rate, ultrasonic velocity, interfacial bond strength and bond slip). Through the observation and study of concrete that is close to the central point of the steel bar, using a scanning electron microscope, the relationship between porosity, the most probable pore diameter and bond strength is established. In addition, we also offer a model for the bond strength damage under the joint actions of FTCs and SBC, and the influences of FTCs and SBC on the bond strength between the reinforcement and concrete are quantitatively analyzed. The test results provide supporting data and a reference for the evaluation of the durability and structural safety of concrete structures in cold environments.

## 2. Materials and Methods

**Figure 1:**
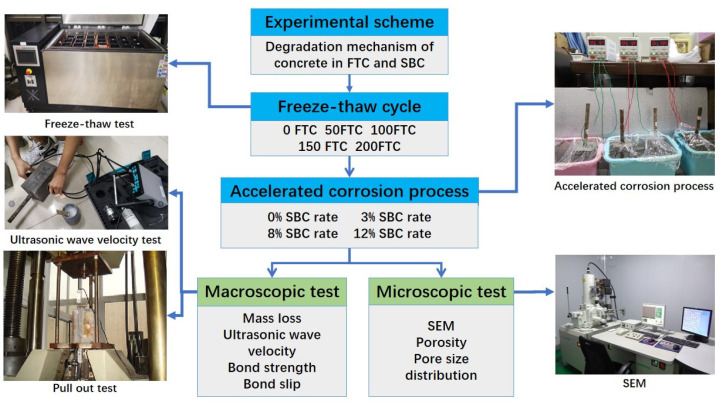
Flow diagram of the experiment.

The experimental program aimed to test the bond strength between the steel bars and concrete with different degrees of corrosion following FTCs and to explore how the FTCs and SBC influenced the bond strength. A series of orthogonal tests with different FTCs and different SBC rates were designed. Firstly, the samples were placed under different FTCs: F0 (0 FTC), F50 (50 FTC), F100 (100 FTC), F150 (150 FTC) and F200 (200 FTC). Different electrochemical-accelerated corrosion processes were then conducted for X0 (uncorroded), X1 (3% theoretical corrosion rate), X2 (8% theoretical corrosion rate) and X3 (12% theoretical corrosion rate). The experimental procedure included the FTC process, accelerated corrosion process and central pullout process. The macroscopic parameters tested included the corrosion quality, ultrasonic wave velocity, bond strength and bond slip. The microscopic parameters tested included the porosity and pore size distribution (PSD). The general test procedure is shown in Figure 1.

### 2.1. Materials

The cement used was ordinary Portland cement P. O 42.5, produced by the Hubei Cement Plant, Yichang, China. Sand with a fineness modulus of 2.78 was used as the fine aggregate. The coarse aggregate was crushed stone with diameters ranging from 5 to 40 mm. Yichang Building Materials Co., Ltd., Yichang, China. provided the steel bars and class I fly ash for this experiment. The high-efficiency water-reducing agent and air-entraining agent were provided by Shanxi building materials Co., Ltd., Weinan, China. Tap water was used in all the mixtures. Deformed steel bars with a diameter, yield strength and elastic modulus of 20 mm, 568 MPa and 200 GPa, respectively, were used to prepare the pullout specimens. The materials used in this experiment were all from China. The mix proportions and performance of the concrete are shown in Table 1.

### 2.2. Specimen Preparation

**Figure 2:**
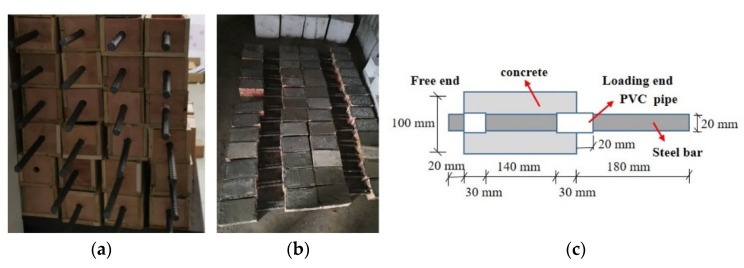
Fabrication of the specimens: (**a**) Wood mold preparation, (**b**) Pouring and maintenance, (**c**) Specimens for pull-out tests.

Pullout tests were performed using a cuboid concrete specimen with a side length of 100 mm × 100 mm × 200 mm and a concentric steel bar placement to investigate the bond behavior between the concrete and the deformed steel bars after the FTC. The number of FTCs and SBC rate were used as variables in the experiment. Three equal specimens were drawn for each condition, giving a total of 60 specimens. The length of the bond anchorage zone was 7 d (140 mm), and the bond zone was established in the middle of the specimen. Simultaneously, the unbonded section was set up to eliminate the local extrusion effect at the loading end. The steel bar had an unbonded section at the loading and free ends. To ensure that the bond stress distribution was more uniform and to avoid local damage at the loading end, the steel bar in the unbonded section was covered with PVC plastic pipe. In addition, after installing the steel bar, the casing was filled using the melted paraffin method to prevent the cement paste from entering the casing when the concrete was poured and to ensure the length of the bond between the steel bar and concrete. The free end of the PVC pipe was filled with wax, and the specimen was soaked in water before freezing and thawing to avoid contact between the steel bar and water. The exposed steel bar was coated with glycerol to prevent corrosion, and the specimen was placed in the standard maintenance room after the molds had been removed. The Chinese standards, namely, the Design Code for Concrete-Face Rockfill Dams [25] and “Steel for the Reinforcement of Concrete—Part 2: Hot-Rolled Ribbed Bars”, were referred to for the specimen preparation. The flow chart for the fabrication of the specimens is shown in Figure 2.

### 2.3. Freeze–Thaw Cycle Test

After casting, the specimens were removed from the molds after 24 h and then moved into the standard maintenance room and kept at a temperature of 20 ± 3 °C and 95% relative humidity for 24 days. The specimens were immersed in water for 4 days before the freeze–thaw cycle tests were conducted in order to ensure that the concrete was water-saturated. The FTC tests were performed using a freeze-thaw machine (type HDK-9) following the Chinese code GB/T 50082-2009 [26]. In each FTC, the temperature of the control specimen decreased from 8 ± 2 °C to −17 ± 2 °C and then increased to 8 °C ± 2 °C within 4 h. After every 50 FTCs, the specimen was removed in order to be photographed and tested for the mass and ultrasonic velocity. When some of the specimens reached the preset number of FTCs, they were removed from the freeze-thaw laboratory and prepared for the accelerated corrosion tests.

### 2.4. Accelerated Corrosion Process

The accelerated corrosion testing of the steel bars began after the end of the FTC tests. According to the grouping, each group of FTC tests included four corrosion states, and 60 specimens were subjected to electrochemical migration acceleration tests with different degrees of corrosion. The full immersion method was used in this experiment to eliminate the effect of humidity on the concrete resistance and the chloride ion transfer rate [27]. In general, electrochemical-accelerated corrosion accelerates the chloride ion corrosion process and ensures the accuracy of the test results [28]. In the electrochemical-accelerated corrosion test, the steel bar in the concrete was selected as the anode of the electrolytic cell, the copper bar was used as the cathode, and a wire was welded to the middle of the steel bar for the application of the current. An SS2323 tracking DC stable power supply was used, and the current used for accelerating the corrosion was 0.2 A. After the test, the condition of the circuit was checked daily. If the copper sheet was consumed or covered by rust, it was cleaned and replaced.

### 2.5. Pullout Test

To investigate the effects of the FTCs and SBC on the bond performance between the steel bar and concrete, the bond strength and bond slip were measured using the central pullout test, in accordance with the Chinese Code of Standard for Test Methods for Concrete Structures GB/T 50152-2012 [29]. All the pullout tests were performed during the same period. The loading machine was a WAW-Y1000C universal testing machine, which was used for the pullout load of the loading end of the steel bar, and data relating to the test pullout force and time were obtained. The maximum load value of the preset load was 200 KN, and the load test was divided into three steps: first, the test force was raised at an average speed of 0.05 KN/s until it reached 3 KN; then, the test force was decreased at an average speed of 0.05 KN/s until it reached 1 KN; and finally, the test force was increased at a constant rate of 0.05 KN/s until the specimen was destroyed. In the test, a crystal quasi CW341 displacement meter with an accuracy of 0.001 m was used to measure the slip at the loading end. Two displacement meters were symmetrically placed on both sides of the concrete loading end (epoxy resin was used for leveling when the concrete surface was uneven) to obtain the slip value of the loading end during the whole process, from loading to the bond failure.

### 2.6. Ultrasonic Wave Velocity Test

The ultrasonic wave velocity test is a non-destructive testing method used to obtain the strength and defects of concrete by measuring the change in the ultrasonic wave velocity in the concrete. It can accurately reflect changes in the internal damage of the concrete after the freezing and thawing cycle. Our test used the Pundit Lab ultrasonic detector (Proceq, Schwerzenbach, Switzerland) to measure the ultrasonic wave velocity of the reinforced concrete specimens. Before the instrument was used, a special coupling agent was applied to the end of the transducer, and it was calibrated with a calibration rod. Then, the sensor frequency, test path, width and calibration coefficient were set according to the specification requirements. Before the experiment, the couplant was also applied to the measuring position on the surface of the test piece, and the ultrasonic wave velocity of the test piece was measured using the counter measurement method.

### 2.7. Microscopic Test

The macroscopic mechanical properties of concrete, such as the bond strength and bond slip between the steel bar and concrete, are determined by its internal microstructure. The internal pore structure of concrete, in particular the porosity and pore size distribution, affects the macroscopic mechanical properties of concrete. After the central pullout test, representative samples with a thickness, length and width of 1 mm, 3 mm and 3 mm, respectively, were collected from the bonding area between the reinforcement and the concrete using a diamond saw. A JEOL JSM 7500F scanning electron microscope (SEM) was used to analyze the microstructural arrangements of the C-S-H and C–H gels and other hydrated products in concrete under the joint actions of the FTCs and SBC. Before the SEM test, the concrete samples were sprayed with high-pressure gold to obtain high-quality images. The images were processed and analyzed based on different grey level values using Image-Pro Plus V6.0 (Media Cybernetics Image Technology Co., Ltd., Rockville, MD, USA) [30,31] to determine the porosity and pore size distribution.

## 3. Results and Discussion

### 3.1. Results

#### 3.1.1. Failure Modes

**Figure 3:**
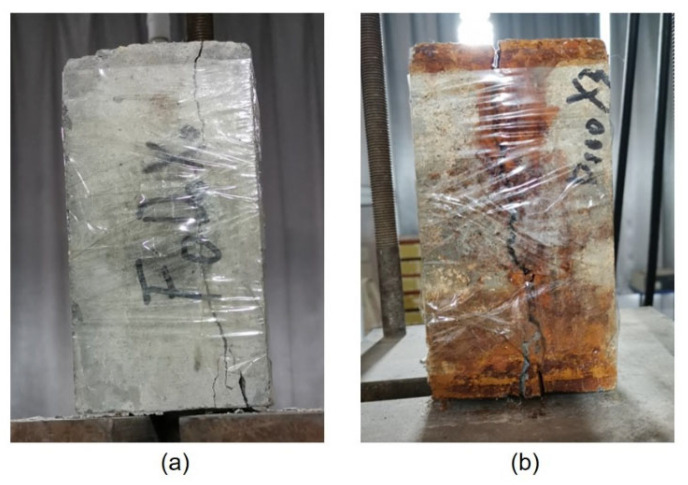
Splitting failure: (**a**) 3% corrosion rate with 0 FTC, (**b**) 8% corrosion rate with 100 FTCs.

Under the designed FTC load, loading tests of the pullout specimens with different rates of SBC were conducted, and the entire bond failure process was studied. Two bond failure modes, i.e., the splitting and pullout failures, were noted. The pullout test results showed that, before 100 FTCs, the specimens suffered total split failure, the splitting failure shown in Figure 3. The cracks first appeared in the covered concrete near the loading end of the specimen and then extended to the covered concrete close to the free end. As can be seen from Figure 3, the crack extended from the loading end to the free end and, subsequently, the crack expanded throughout the specimen, the covered concrete split completely and the test ended. It was observed that 100 FTCs did not change the mode of the bond failure between the corroded steel bar and concrete. After 150 and 200 FTCs, the specimens with corrosion rates of 8% and 12% were pulled out. Thus, 150 FTCs and an 8% corrosion rate may be the critical points of the two failure modes.

#### 3.1.2. Mass Loss and Ultrasonic Wave Velocity

During the FTC process, the concrete surface gradually transformed from compact to porous, which induced a decrease in the mass of the specimens. The mass loss can be used to quantify the spalling of concrete after FTCs. The corrosion products caused by SBC also led to changes in the quality of the specimens. To study the effects of different FTC and SBC rates on the mass loss rate, the effects of the FTCs on the mass loss rate under the same corrosion degree were compared, and the relationships between the material quality and corrosion rate with the same number of FTCs were also compared.

**Figure 4:**
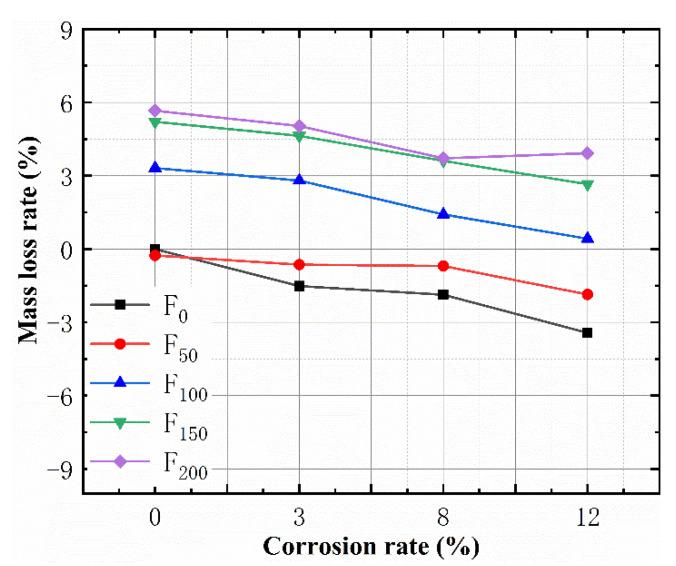
Relationship between the corrosion rate and mass loss rate.

In Figure 4, positive and negative values represent a decrease and increase in the mass, respectively. The mass loss rate decreased with the increase in the theoretical corrosion rate (Figure 4). The mass loss rate increased with the FTC increase. The greater the amount of corrosion was, the more corrosion products were produced, while the quality increased and the mass loss rate decreased. The greater the number of FTCs was, the more the concrete quality decreased and the mass loss rate increased.

For the uncorroded experimental group, the mass loss rate was negative before 50 FTCs. This is because, at the beginning of the FTCs, the microcracks caused by the freeze-thaw damage do not cause the concrete to peel off but allow water to enter the concrete and increase the total mass. After 100 FTCs, the mass loss rates of F100, F150 and F200 concrete specimens were 3.32%, 5.21% and 5.66%, respectively. It can be seen that the mass loss rate of the specimens increased as the FTCs increased. It can also be concluded from Figure 4 that, for the specimens with a theoretical corrosion degree of 3%, the mass loss of specimens in the 50 FTCs group was unchanged, indicating that the quality increase by the 3% corrosion was the same as the quality reduction by 50 FTCs. The corrosion products produced by the 3% corrosion filled the pores of the concrete and enhanced the freeze–thaw resistance. At 200 FTCs, the mass loss rate increased slightly when the theoretical corrosion was >8%, indicating that the increased quality of corrosion was less than the reduced freeze–thaw mass, and the excessive SBC rate accelerated the damage to the concrete caused by the FTCs.

**Figure 5:**
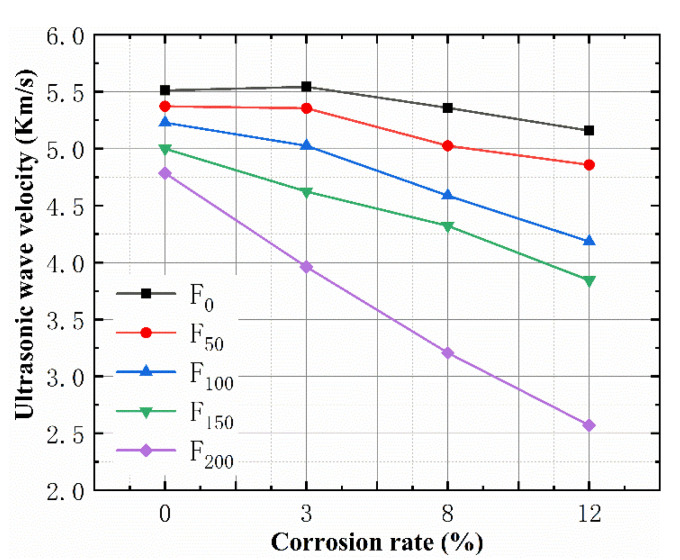
Relationship between the corrosion rate and ultrasonic wave velocity.

Figure 5 shows the relationship between different SBC rates and the ultrasonic velocity after the FTCs. It shows that the ultrasonic wave velocity decreased with the increase in the FTCs and linearly decreased with the increase in the corrosion rate. For the specimens subjected to 0 and 50 FTCs, the ultrasonic wave velocity decreased sharply when the SBC rate was >3%. When the SBC rate was ≤3%, the ultrasonic wave velocity of the specimens subjected to 50 FTCs was unchanged, but the specimens subjected to 0 FTCs slightly increased. This is consistent with the change law of the mass loss rate, because the reinforced concrete forms a layer of rust on the bond surface between the steel bar and the concrete after mild corrosion, and the volume becomes larger because the density of rust is very small. Corrosion products fill the pores of the concrete, and the sample becomes denser, which improves the ultrasonic wave velocity. With a continuous increase in the SBC rate, the excessive volume of the corrosion products leads to cracks in a concrete specimen, resulting in a reduction in the ultrasonic wave velocity.

#### 3.1.3. Bond Strength and Bond Slip

Bond stress τ is defined as the average shear stress along the buried length between the steel bar and the surrounding concrete. Based on the pullout test results, the bond stress of the steel bars in the specimens was constant with the anchorage length. Hence, an average bond strength *τ* can be calculated as follows:

(1) $$\tau =\frac{P_{}}{\pi dl_{a}}$$

where *τ* is the average bond strength (in megapascals) and *P* is the pullout force of the steel bar (when *P* reaches the peak pullout force, τ is also termed as the bond strength). In Newtons, *d* is the diameter of the steel bar (in millimeters) and $l_{a}$ is the bond length (in millimeters). By calculating the average values of the test results of the three specimens, the pullout force *P*, bond strength *τ* and bond slip *s* corresponding to bond strength were calculated. Figure 5 shows the relationship between the bond strength and the corrosion rate.

**Figure 6:**
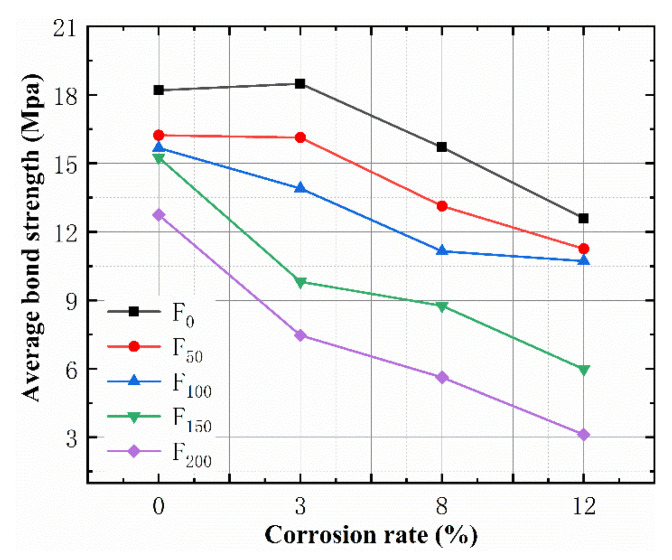
The relationship between the bond strength and corrosion rate.

Figure 6 shows that the corrosion rate had a significant influence on the bond strength, and the average bond strength decreased as the corrosion rate increased. However, when the corrosion rate was 3%, the average bond strength of the not subjected to the freeze–thaw cycle increased by a small margin, and the bond strength of the specimens after 50 FTCs did not significantly decrease. This is mainly because, in the case of a low corrosion rate, the micro-corrosion products fill the pores in the bond section of reinforced concrete, and the corrosion products increase the surface roughness of the steel bars, thus increasing the friction between the steel bars and concrete. Similarly, the expansion volume of the corrosion products was about two to six times greater than it was before the corrosion, which squeezed the concrete around the steel bar, thus increasing the friction between the concrete and the steel bar and increasing the bond strength. When the corrosion rate was >3%, the corrosion products increased, and their increased volume produced extrusion pressure on the concrete, resulting in microcracks on the concrete around the steel bar, thus reducing the bond strength between the steel bar and the concrete.

Figure 6 also shows that the bond strength of the concrete specimens decreased as the FTCs increased, and the freeze-thaw damage reduced the bond performance between the concrete and steel bars. Repeated FTC damage led to further expansion of the cracks, weakened the effective restraint of the concrete around its reinforcement to the steel bar, and reduced the bond strength. With the aggravation of freeze-thaw damage, the cracks caused by the FTCs extended from the concrete surface to the steel bar, accelerating the corrosion rate of the steel bar. The interaction between SBC and freeze-thaw cracks led to the accelerated rate of damage of the bond strength. It can be seen from the slope of the relationship between the bond strength and the corrosion rate that the rate of loss of the specimen’s bond strength increased after 100 FTCs, and the rate of the decrease in the bond strength was fastest after 150 FTCs and 200 FTCs, which indicated that the damage of the bond strength was mainly caused by the FTCs at this time.

**Figure 7:**
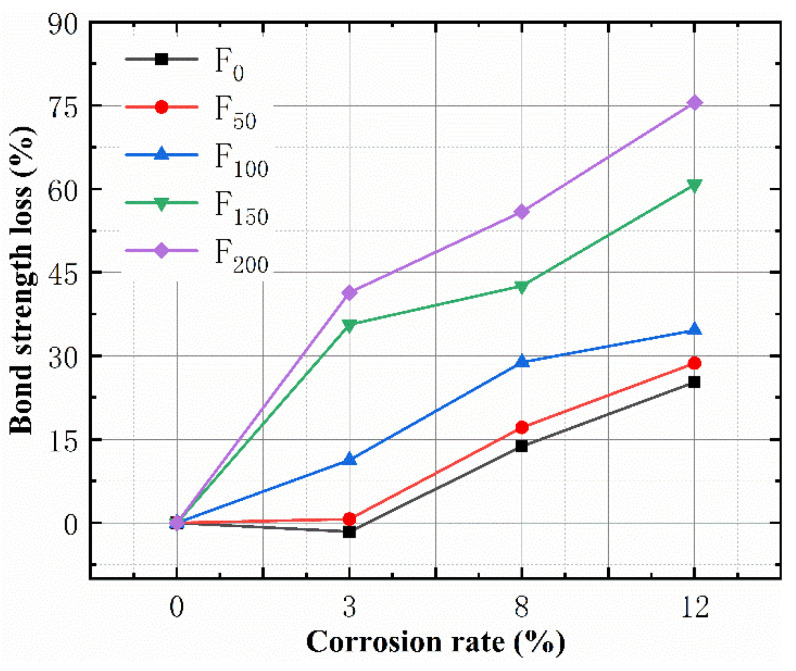
The relationship between the bond strength loss and corrosion rate.

A comparison of the 0 FTC specimens showed that the bond strength loss of all the specimens could be obtained. Figure 7 shows the bond strength loss of the specimen after the joint actions of the FTCs and SBC. The loss rate of the bond strength increased with the number of FTCs, and the 150 FTC and 200 FTC specimens showed the most rapid loss of bond strength. For samples with corrosion rates of ≤3%, the loss rate of the bond strength of the 0 FTC samples was negative, because the corrosion increased the bond strength. After 50 FTCs, the loss rate of the bond strength of the samples with a corrosion rate of 3% did not change, indicating that the damage caused by the FTCs was alleviated by this time. This is similar to the results of the mass loss rate and ultrasonic speed loss tests. A corrosion rate of ≤3% increased the bond strength and offset the loss of bond strength caused by 50 FTCs.

**Figure 8:**
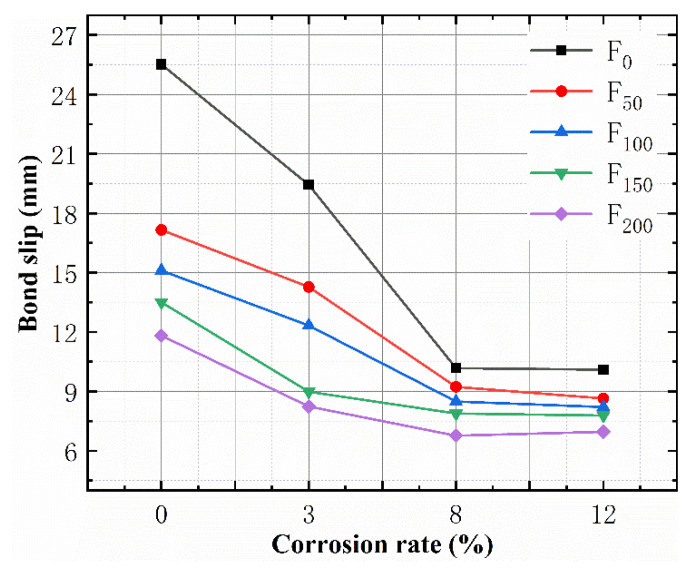
The relationship between the bond slip and corrosion rate.

The bond slip behavior is the main evaluative indicator of the structural safety of reinforced concrete. The bond slip behavior of the concrete specimens under the joint actions of the FTCs and SBC is shown in Figure 7. The FTCs had a significant effect on the bond- lip performance of the reinforced concrete, and the maximum bond slip decreased as the number of FTCs increased. For the uncorroded specimens, the bond slip decreased by 53.68% after 200 FTCs, and the largest decrease in the bond slip occurred after 50 FTCs, reaching 32.76%, indicating that 50 FTCs had a significant influence on the bond slip and may have destroyed the chemical bond between the steel and concrete. In addition, Figure 8 shows that the bond slip decreased as the corrosion rate increased, but it remained unchanged after the theoretical corrosion ratio reached 8%. Under the FTC action, the theoretical corrosion rate of <8% reduced the slip between the steel bar and concrete, worsened the ductility and caused the slip state to appear earlier. The fastest rate of the reduction of the bond slip was observed in the uncorroded specimens with a corrosion rate of less than 8%, whereas the slowest rate was observed in the 150 FTC and 200 FTC specimens. A theoretical corrosion rate greater than 8% had little effect on the bond slip.

#### 3.1.4. Microstructure Evolutions

##### Micromorphology

**Figure 9:**
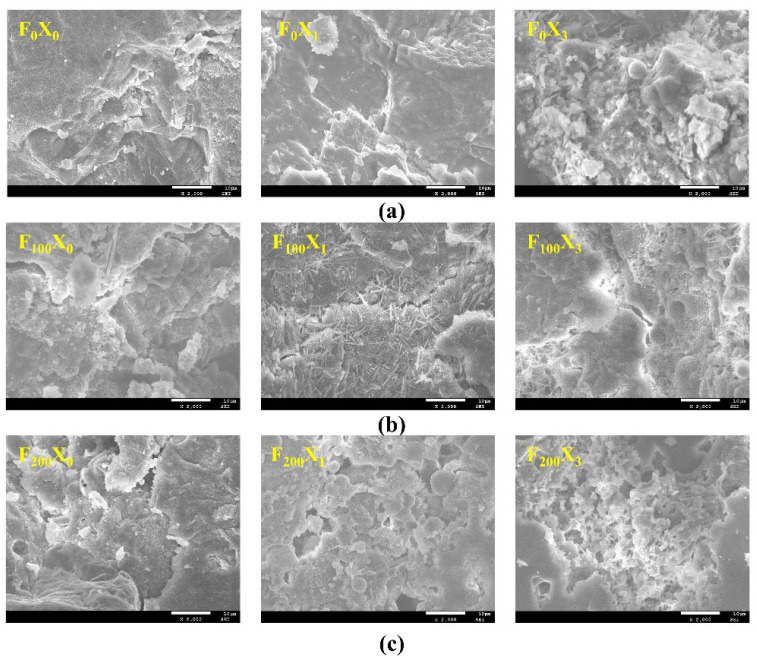
Electron microscope scanning images of different corrosion rate groups subjected to FTCs: (**a**) different corrosion rates with 0 FTC, (**b**) different corrosion rates with 100 FTCs, (**c**) different corrosion rates with 200 FTCs.

According to the abovementioned macroscopic test results, FTCs and SBC have significant effects on the bond properties of reinforced concrete. Nine experimental groups with FTCs (0, 100 and 200) and SBC rates (0%, 3% and 12%) were selected for the comparison of the scanning electron microscope images before and after corrosion in order to further explore the influences of FTCs and SBC on the bond performance between steel bars and concrete from a microscopic point of view. The pore structures of the specimens with the three corrosion rates subjected to the actions of the FTCs are shown in Figure 9 (2000× magnification).

Figure 9a shows that, after 28 days of curing, the cement within the concrete showed complete hydration, with a smooth and dense accumulation of hydrated calcium silicate, a smooth and complete structure and no cracks. In addition, in the specimens subjected to a 3% corrosion rate, the corrosion product filled the pores of the hardened cement paste, the structure was more compact and the porosity was reduced. The specimens with a 12% corrosion rate showed spherical particles and some holes and cracks. This is due to the high corrosion rate and the formation of a large number of Fe_2_O_3_ products. Because of the large volume of Fe_2_O_3_ and the expansion force acting on the surrounding cement materials, microcracks were observed in the structure, with an increased porosity. Figure 9b shows that, for the specimens with 100 FTCs, the internal concrete microstructure changed slightly, and the dense packing state before freezing and thawing gradually became loose and cracked. This is due to the degenerated gelling property, weakening of the interaction between the hydrated calcium silicate gels, and a decrease in the adhesion between particles after repeated FTCs. The generation of these cracks had an adverse impact on the overall structural performance. As can be seen from Figure 9c, for the specimens with 200 FTCs, the internal concrete microstructure significantly changed. Compared with the 0 FTC specimens and 100 FTC specimens, the sample structure became loose and honeycombed, and large cracks could be observed. With increasing freeze–thaw cycles, some small cracks continued to expand and connect, forming large cracks, which had a serious impact on the material structure.

Generally speaking, the microscopic properties of concrete changed with increasing freeze–thaw cycles, and the mechanical properties of the concrete matrix decreased, leading to a poor bonding performance. With a similar corrosion rate, the structure became continuously looser as the FTCs increased, indicating that the damage to the microstructure caused by FTCs provides a channel for the transport of water and ions and accelerates the formation of corrosion products. In summary, under the combined actions of the FTCs and SBC, the deterioration process of the concrete involved the expansion of a large number of macropores and microcracks. With the development and expansion of microcracks caused by the joint actions of the FTCs and SBC, the internal structure of the concrete specimens was no longer compact. Therefore, the periodic FTCs and excessive corrosion rates produced a large number of harmful pores in the concrete, which greatly reduced its durability. Additionally, the ultrasonic wave velocity and mechanical properties of the concrete gradually decreased.

##### Porosity Analysis

**Figure 10:**
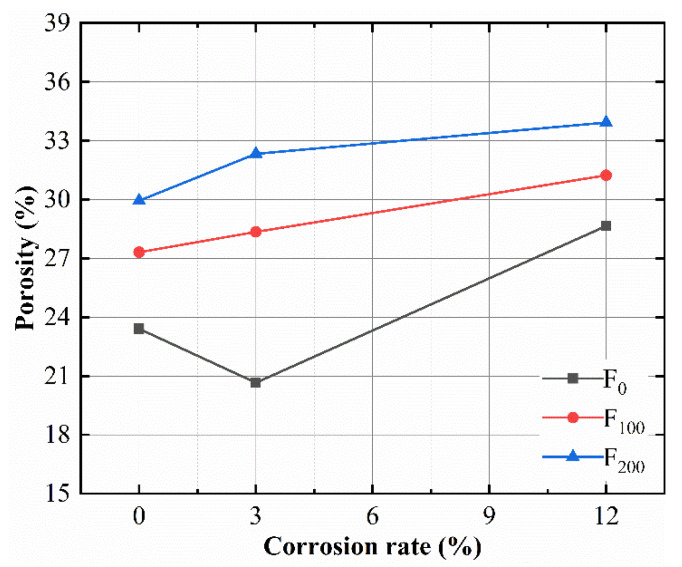
The effect of the corrosion rate on the porosity of specimens.

The 0, 100 and 200 FTC samples were selected for the analysis of the change law of the porosity close to the steel bar at different SBC rates combined with FTC in order to further quantify the relationship between the microstructure and macroscopic properties. In addition, the influences of the FTC and SBC rates on the bond strength was explained from a microscopic point of view. The relationship between the corrosion rate and porosity after the FTCs is shown in Figure 10.

Figure 10 shows that the porosity increased with increased FTCs. For the 0 FTC specimens, the porosity decreased, at first, and then rapidly increased with the increase in the corrosion rate. Under the action of a corrosion rate of ≤3%, the corrosion product filled the pores and reduced the porosity. As the corrosion rate increased, the extrusion pressure caused by too many corrosion products exceeded the tensile strength of the concrete, resulting in cracks in the concrete and an increase in the porosity. For the 100 FTC specimens, the porosity increased linearly with the increase in the corrosion rate. This showed that the concrete had been damaged by the freeze-thaw at 100 FTCs, that 3% of the corrosion products was insufficient to fill the cracks caused by the freeze-thaw, and that the FTCs and SBC had caused superimposed damage to the concrete. Compared with the 0 and 200 FTC samples with a corrosion rate of 12%, the porosity of the 200 FTC samples increased by 10.5%, while that of the 0 FTC samples increased by 4.9%, which indicates that the increase in the porosity caused by SBC with a 12% corrosion rate was less than that caused by 200 FTCs. 

##### Pore Structure

The pores in concrete are formed by cement hydration, and they are distributed in the cement mortar. The porosity, PSD and pore connectivity affect the transport properties of water and chloride ions in concrete, as well as its mechanical properties. Of these, PSD is the best method for evaluating the comprehensive properties of hydrated cement paste. Under the actions of 0, 100 and 200 FTCs, the specimens with the three corrosion rates of 0%, 3% and 12% were selected as samples for the PSD analysis.

**Figure 11:**
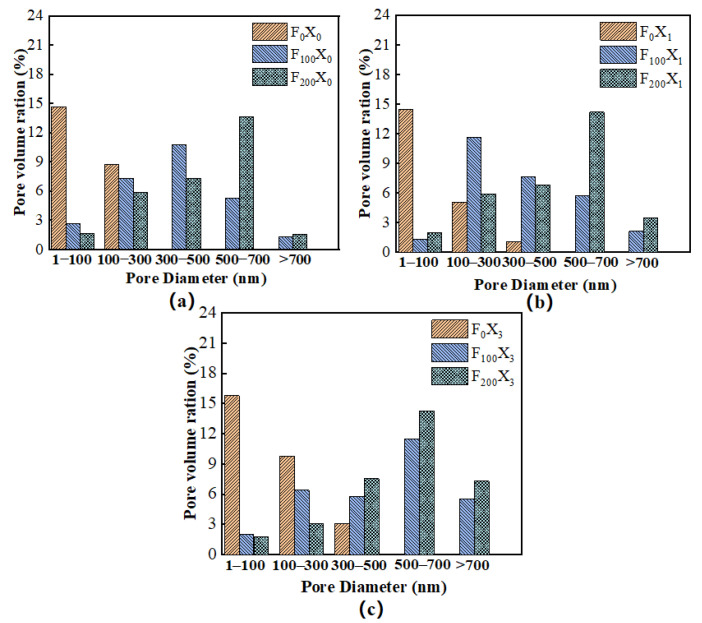
Pore size distribution of the specimens with different corrosion rates: (**a**) uncorroded group, (**b**) 3% corrosion group, and (**c**) 12% corrosion group.

Figure 11 shows the PSD results of the specimens with different SBC rates after FTCs. Since the most probable pore diameter represents the pores with the greatest degree of porosity, Figure 11 shows that the most probable pore diameter increased as the number of FTCs increased at similar corrosion rates. FTC damage led to an increase in the most probable pore diameter and the porosity of the concrete, a decrease in the concrete strength and a decline in the bonding performance. With the same number of FTCs, the most probable pore diameter was also related to the corrosion rate. Under the actions of the three corrosion rates, the most probable pore size of the 0 FTC sample was <100 nm. After 100 FTCs, the most probable pore diameters of three specimens were 300–500, 100–300 and 500–700 nm, which indicated that the corrosion products with a 3% corrosion rate (X1) filled part of the pores of diameters of 300–500 nm at 100 FTC, thus reducing the most probable pore diameter. In addition, the corrosion products with a 12% corrosion rate (X3) led to an increase in the most probable pore diameter.

Figure 10 shows that the number of pore sized <100 nm was unchanged for the 0 FTC specimens. After 200 FTCs, the number of pores sized >500 nm increased sharply. In addition, damage after 200 FTCs led to cracks in the pores sized <500 nm, and connected macropores were formed, which eventually increased the number of pores of >500 nm. Due to the repeated accumulation of hydrostatic pressure, the expansion force and other destructive effects during the freeze–thaw cycle, the internal damage of the concrete gradually increases, and the pores and microcracks also continued to expand and gradually developed into interconnected large cracks, eventually leading to the increase in the internal pore diameter and porosity of the concrete. The porosity and macropore ratio in the concrete increased as the number of FTCs increased, which is the main reason for the decrease in the bond strength with the increase in the porosity, consistent with the results of Zhang et al. [32].

### 3.2. Discussion

Based on a fitting analysis of the meso- and macroscopic parameters of the concrete specimens with different reinforcement corrosion rates under freeze-thaw conditions, the degradation mechanism of the bond strength under the combined actions of the FTCs and SBC was considered. The relationship between the pore structure and bond strength was evaluated, and the influenced of the FTCs and SBC on the bond strength of the concrete is discussed below.

#### 3.2.1. Effect Mechanism

Chloride penetration is the major cause of steel corrosion in reinforced concrete. From the abovementioned micromorphology analyses combined with the macroscopic strength degradation law, on the one hand, we can observe that FTCs change the internal structure of concrete and the chemical adsorption between the cement gel and steel bar surface, and may weaken the friction between the concrete and steel bar interface, which reduces the bond strength between the concrete and steel bar. On the other hand, the increase in the number of macropores and microcracks caused by FTCs provides more channels for chloride ion penetration and accelerates the steel bar corrosion. With increasing volume, the corrosion products produce a squeezing pressure on the concrete around the steel bar, which causes the cracks to expand further. The FTCs not only increase the most probable pore diameter, but also increases the transport performance of the water and chloride ions in the concrete, making it easier for external oxygen to come into contact with the steel bar. Under the combined actions of FTCs and SBC, the bond strength between the steel bar and concrete decreases, and the bond performance deteriorates.

At the microlevel, freeze-thaw damage changes the number of connected holes and water transport performance in concrete. These pores affect the microstructure distribution of the concrete and accelerate the migration of the pore water. In addition, more water becomes ice, and volume expansion produces tensile stress, resulting in new microcracks. Thus, the bond strength is reduced. The generation of new cracks creates favorable conditions for corrosion and produces more corrosion products, while the corrosion products of the volume expansion also produce tensile stress on the concrete, and the damage of the FTCs and SBC on the bond strength of the concrete therefore occurs as a cumulative effect.

#### 3.2.2. Relationship between the Pore Structure and Bond Strength

**Figure 12:**
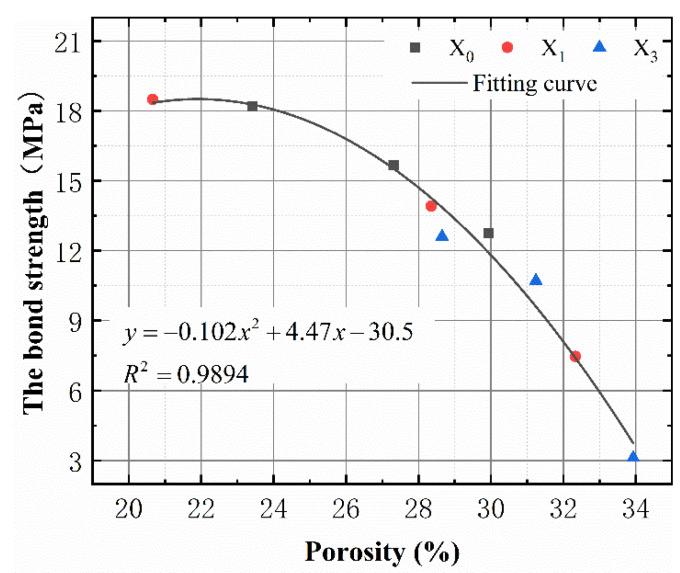
The relationship between the porosity and bond strength.

**Figure 13:**
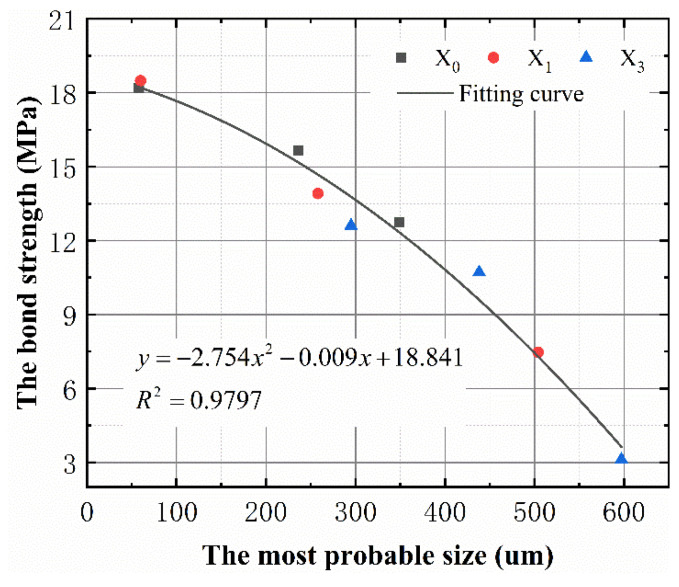
The relationship between the most probable pore size and bond strength.

The relationship between the bond strength and pore structure of the specimens with different corrosion rates under the action of the FTCs was analyzed in order to study the effect of the FTCs on the bond strength between the steel bars and concrete. The relationship between the bond strength and porosity of concrete specimens with different corrosion rates under the action of the FTCs is shown in Figure 12 and Figure 13. In addition, Figure 11 shows the relationship between the bond strength and porosity in the 200 FTC specimens subjected to different corrosion rates. Irrespective of the corrosion rate of a steel bar, a nonlinear negative correlation exists between the bond strength and porosity. The bond strength decreases as the porosity increases. From the microstructure point of view, the increase in the porosity leads to an increase in the water transport channels and accelerates the freeze-thaw damage. The freeze-thaw damage provides transport channels for the ions and accelerates the SBC.

Considering that the most probable pore diameter represents the greatest degree of total porosity, Figure 13 shows the relationship between the most probable pore diameter and bond strength under the influence of the FTCs. It shows that the most probable pore diameter of the bond strength between the steel bar and concrete under the influence of the FTCs had a nonlinear negative correlation. Thus, the correlation coefficient is 0.9797, which reflects a good correlation. Figure 13 shows that the bond strength decreased as the most probable pore size increased.

#### 3.2.3. Bond Strength Damage Model

According to the above analysis, the porosity of concrete is related to the number of FTCs and the SBC rate. Based on multiple regression analysis, the model of the relationship between the porosity and the number of FTCs and the SBC rate was established, and it can be expressed as follows:

(2) P=3.92n+0.3894c+22.5565

where *n* is the number of FTCs divided by 100, *P* is the porosity of concrete after the FTCs and *C* is the SBC rate. Equation (2) shows that the porosity decreases as the FTC and SBC rates increase. Substituting Equation (2) into the model of the relationship between the bond strength and porosity in Figure 11, the degradation model of the relationship between the bond strength and FTC and SBC rates can be obtained as follows:

(3) $$\tau =-1.57n^{2}-0.0155c^{2}-0.312nc-0.516n-0.051c+18.43$$

where *τ* is the average bond strength (in megapascals). Substituting the data measured in the laboratory into Equation (3), the results are consistent, and the error is small, which shows that the model can effectively predict the damage rate of the bond strength according to the number of FTCs and SBC rates. The bond strength damage model also shows that the effect of the FTCs on the bond strength is slightly greater than that of the SBC rate.

#### 3.2.4. Relativity Analysis

Correlation refers to the degree and direction in which two variables are related. Pearson′s relationship coefficient is the covariance of two factors separated by the result of their standard deviations [33], and it is a measure of association existing between two variables. Pearson’s product-moment correlation model was used for this analysis, it is expressed as [34]:

(4) $$r=\frac{\sum \left(x-\bar{x}\right)\left(y-\bar{y}\right)}{\sqrt{\sum {\left(x-\bar{x}\right)}^{2}}\sqrt{\sum {\left(y-\bar{y}\right)}^{2}}}$$

where *r* is Pearson′s coefficient of correlation, x,y are the independent value and $\bar{x},\bar{y}$ are the average value. The value of Pearson’s coefficient of correlation “*r*” lies between +1 and −1. Positive (+) values of “*r*” mean that there is a “direct correlation,” and negative (−) values of “*r*” mean that there is an “inverse correlation”. The Pearson model was used to analyze the bond strength, ultrasonic velocity and porosity data under different FTC and SBC rates in order to judge their compactness and study the relationship between the number of FTCs and SBC on macro-strength and micro-index. The results of the correlation analysis are shown in Table 2.

The results in Table 2 show that a significant correlation exists between the bond strength and the FTCs, corrosion rate, ultrasonic speed and porosity, with correlation coefficients of −0.749, −0.606, 0.923 and −0.945, respectively. A positive correlation exists between the bond strength and ultrasonic speed, and three other negative correlations were noted. The correlation coefficient between the porosity and bond strength is the largest, reaching −0.945, showing a good correlation between the porosity and bond strength. The correlation coefficients between the porosity and the FTCs, corrosion rate and ultrasonic velocity are 0.801, 0.5 and 0.923, respectively, indicating that porosity is positively correlated with the number of FTCs and negatively correlated with the ultrasonic velocity. The correlation coefficient between the FTCs and the theoretical corrosion rate is 0, indicating no correlation. From the comparison of the correlation coefficients, the influence of the FTCs on the bond strength is shown to be greater than that of the corrosion rate.

## 4. Conclusions

Through the accelerated corrosion testing of different steel bar corrosion (SBC) rates under freeze–thaw cycle (FTCs) conditions, the effects of FTCs and SBC on the bond strength between the steel bar and concrete were studied, and the evolution rules of the effects of the FTCs and SBC on the macroscopic strength and concrete microstructure were obtained. The main conclusions are summarized as follows:

(1) The effect of FTCs on corrosion is a process varying from micro-quantitative alterations to macro-qualitative alterations. The micro-damage of the cement paste caused by FTCs is cumulative and reduces the bond strength directly. Moreover, the micro-damage can also increase the transport process of ions and, then, accelerate the corrosion.
(2) The corrosion products, at a lower corrosion rate (≤3%), fill a portion of the pores of the concrete and increase the friction between the steel bar and concrete. Then, it can alleviate the damage caused by 50 FTCs to an extent. The bond slip decreases as the corrosion rate increases but is almost unchanged when the corrosion rate exceeds 8%.
(3) The relationship between the macro-mechanical properties and micro-pore structure shows that freeze-thaw damage increases the porosity and the most probable pore diameter of concrete, leads to a decrease in the concrete strength and reduces the bond strength. Freeze-thaw can also accelerate the corrosion rate, reducing the bond.
(4) The damage model of the bond strength shows that the bond strength decreases with the increasing number of FTCs and SBC rates, and the effect of the FTCs on the bond strength is slightly greater than that of the SBC rate. The results of the correlation analysis also confirm that the effect of FTCs on the bond strength is greater than that of SBC rate.

## Figures and Tables

**Table 1:** The mix proportions and performance of the concrete.

w/c Ratio	Cement (kg/m^3^)	Water (kg/m^3^)	Fly Ash (kg/m^3^)	Sand (kg/m^3^)	Stone (kg/m^3^)	Water-Reducing Agent (%)	Air-Entraining Agent (%)	Cubic Compressive Strength at 28 d (MPa)
0.4	272	136	68	553	1291	1	0.02	35.4

**Table 2:** Calculation results of the correlation coefficient.

	Number of FTCs	Corrosion Rate	Bond Strength	Ultrasonic Wave Velocity	Porosity
Number of FTCs	1	0	−0.749	−0.77	0.801
Corrosion rate	0	1	−0.606	−0.514	0.5
Bond strength	−0.749	−0.606	1	0.923	−0.945
Ultrasonic wave Velocity	−0.77	−0.514	0.923	1	−0.835
Porosity	0.801	0.5	−0.945	−0.835	1

## Data Availability

Not applicable.
